# Neuroprotective Effect of Physical Activity in Ischemic Stroke: Focus on the Neurovascular Unit

**DOI:** 10.3389/fncel.2022.860573

**Published:** 2022-03-04

**Authors:** Hui Zhang, Qi Xie, Juan Hu

**Affiliations:** ^1^School of Physical Education, Nanchang University, Nanchang, China; ^2^Inpatient Department, Jiangxi Provincial People’s Hospital, Nanchang, China; ^3^Yu Quan dao Health Center, Jiangxi Provincial People’s Hospital, Nanchang, China

**Keywords:** exercise, cerebral ischemia, neurovascular unit, blood-brain barrier, mechanism

## Abstract

Cerebral ischemia is one of the major diseases associated with death or disability among patients. To date, there is a lack of effective treatments, with the exception of thrombolytic therapy that can be administered during the acute phase of ischemic stroke. Cerebral ischemia can cause a variety of pathological changes, including microvascular basal membrane matrix, endothelial cell activation, and astrocyte adhesion, which may affect signal transduction between the microvessels and neurons. Therefore, researchers put forward the concept of neurovascular unit, including neurons, axons, astrocytes, microvasculature (including endothelial cells, basal membrane matrix, and pericyte), and oligodendrocytes. Numerous studies have demonstrated that exercise can produce protective effects in cerebral ischemia, and that exercise may protect the integrity of the blood-brain barrier, promote neovascularization, reduce neuronal apoptosis, and eventually lead to an improvement in neurological function after cerebral ischemia. In this review, we summarized the potential mechanisms on the effect of exercise on cerebral ischemia, by mainly focusing on the neurovascular unit, with the aim of providing a novel therapeutic strategy for future treatment of cerebral ischemia.

## Introduction

Research on neuroprotective therapy for cerebral ischemia has been a hot topic worldwide. Ischemic tolerance refers to a short period when patients experience cerebral ischemia before the onset of a severe ischemic stroke, thus enhancing the tolerance of brain tissue to the subsequent ischemic injury (Wang et al., 2015; Hao et al., 2020; Daniele et al., 2021). This suggests that preischemic interventions can induce tolerance to secondary severe injury after cerebral ischemia. Triggers of various types of mild stressors or stimuli (i.e., surgical, remote ischemic preconditioning, exercise, acupuncture, and pharmacological methods) induce adaptive endogenous tolerance to ischemia injury by activating a multitude cascade of molecules (Thushara Vijayakumar et al., 2016; Vinciguerra et al., 2018; Hao et al., 2020). As a preventive method, exercise is accepted relatively easily by patients. As a safe preintervention approach, exercise actually has less adverse effects, which allows it to be more suitable for clinical application. A large number of studies have demonstrated that exercise before cerebral ischemia, also known as exercise preconditioning, can induce ischemic tolerance, thereby effectively alleviating brain injury and promoting functional recovery after an ischemic stroke (Zhang et al., 2011; Dornbos and Ding, 2012; Sakakima, 2019). Therefore, elucidating the neuroprotective mechanisms and benefits of exercise and exercise intervention, pre- and post-stroke, will encourage patients with high-risk factors of ischemic stroke to actively exercise for prevention and treatment of stroke. The close connection between microvascular endothelial cells and surrounding astrocytes plays an important role in maintaining an intact neurovascular coupling. Regulation of the microvascular basal matrix membrane, activation of endothelial cells, and changes in astrocyte adhesion can directly affect the transmission of information between the microvasculature and neurons.

A neurovascular unit (NVU), which was initially proposed by Lo et al. (2003) in the early 2000s, is a structural and functional unit of the central nervous system. It emphasizes the dynamic interactions among endothelial cells, astrocytes, pericytes, basal membrane, microglia, neurons, and the extracellular matrix and the importance of such interactions in the pathophysiology of stroke (Steliga et al., 2020; Moon et al., 2021; Naranjo et al., 2021; Wang et al., 2021). The concept of NVU not only provides a platform for understanding central nervous system injury, but also provides a possibility for both clinically successful and timely intervention of brain injury (Yu et al., 2020; Zhou et al., 2020). Exercise, as a preconditioning approach, has been discovered to play a neuroprotective role by interfering with NVU (Dornbos and Ding, 2012; Murugesan et al., 2012; Wang et al., 2014; Laitman and John, 2015). This review is a summary of the effect and potential mechanism of exercise on NVU, to provide a theoretical basis for elucidating the neuroprotective mechanism of exercise, and its further application in cerebral ischemia.

## Cerebral Ischemia and Neurovascular Unit Injury

### Cerebral Ischemia and Neuronal Injury

The mechanism of neuronal injury caused by cerebral ischemia is complex. Neuronal apoptosis, after cerebral ischemia, is regulated by many different events and may develop by initiating the internal cellular death mechanism. Many studies have discovered that cerebral ischemia can induce cell apoptosis, and most apoptotic cells are neurons, with a few glial cells and vascular endothelial cells (Chan, 2004; Peng et al., 2004; Radak et al., 2017). Delayed neuronal apoptosis is a process that is different from necrosis, causing neuronal loss and occurring after cerebral ischemia (Huang et al., 2021; Kang et al., 2021). Regional expression of apoptosis-related proteins, such as caspase family, Bcl-2, and Bax, are involved in the occurrence of neuronal apoptosis after the onset of cerebral ischemia (Zhou et al., 2003; Long et al., 2022). It has been reported that brain ischemia for 10 min not only causes neuronal apoptosis in rats, but permanent cerebral ischemia for 60 min causes neuronal apoptosis (O’Sullivan et al., 2007). The number of apoptotic neurons fluctuates in a curvilinear manner over time, indicating that, during the process of cerebral ischemia, the main factors that determine neuronal apoptosis are the severity of ischemia and susceptibility of neurons. Ischemia causes energy failure, leading to increased intracellular sodium and calcium, cell lysis, and neuronal apoptosis. Studies have shown that there are many apoptotic neurons within the ischemic area at 0.5 h after the development of cerebral ischemia (Radak et al., 2017). Apoptotic cells are scattered in the preoptic region, striatum, and cerebral cortex. The number of apoptotic neurons reached a peak at 12 h after reperfusion, and various morphologies of apoptotic cells were observed (Nakka et al., 2008; Li et al., 2017a). The results mentioned above indicate that the sensitivity of neurons to ischemia-reperfusion injury is different, and that neuronal apoptosis is a dynamic developmental process (Naito et al., 2020). The induced expression of many neuronal genes and proteins is initially an adaptive response to cerebral ischemia stress, but due to repeated or continuous occurrence of cerebral ischemia, they gradually alter to promote or inhibit apoptosis, thereby constituting the complex regulatory factor system of neuronal apoptosis within the body (Lehotský et al., 2009; García de la Cadena and Massieu, 2016; Jensen et al., 2019).

Necrosis is another important type of neuronal death. Studies have reported that the activation of receptor-interacting protein kinase (RIPK)-mixed lineage kinase domain-like protein (MLKL) signaling pathway is involved in the onset of neuronal necrosis after injury by cerebral ischemia (Deng et al., 2019; Zhang et al., 2019, 2020). The expression of p-RIPK1 and p-MLKL proteins was found to be increased in the ischemic brain. Furthermore, use of RIPK1 inhibitors could hinder p-RIPK1 and p-MLKL protein expression, with reduced necrosis and infarct volume and promotion of neurological function (Deng et al., 2019; Liao et al., 2020). These data provide evidence that p-RIPK1 is involved in the initiation of RIPK3-/MLKL-induced necroptosis after cerebral ischemia. In addition, Pellino3, which is a ubiquitin E3 ligase, inhibits the formation of the death-induced signaling complex in response to tumor necrosis factor-α (TNF-α) by targeting RIPK1. Furthermore, it has been shown to have an effect in counteracting necroptosis *via* RIPK1 ubiquitination, and neuronal necroptosis could be reduced by upregulation of Pellino3 (Zhang et al., 2021). Zhang et al. (2020) found that knockout of Rip3 or Mlkl had a neuroprotective effect in acute ischemic stroke mice, and that necroptosis loss-of-function mice have attenuated inflammation in the brain infarct tissue. As an efficient and specific inhibitor of RIPK3, Gsk-872 inhibited hypoxia-induced phosphorylation of RIPK1/3 and MLKL proteins, reduced neuronal death, and alleviated brain injury (Yang et al., 2017).

The autophagy signaling pathway in cerebral ischemia is complex, is connected to necrosis and apoptosis, and affects the degree of brain injury (Kim K. A. et al., 2018; Wang et al., 2018; Xu et al., 2021). Su et al. (2014) demonstrated that the autophagy inhibitor 3-methyladenine (3-MA) reversed the neuroprotective effects that were induced by remote ischemic preconditioning against cerebral ischemia-induced injury, whereas the autophagy inducer rapamycin ameliorated neurological dysfunction post-stroke. This indicates that the activation of autophagy is involved in remote ischemic preconditioning-induced neuroprotection in cerebral ischemia (Su et al., 2014). Autophagy activation is common after the onset of brain ischemic/reperfusion injury. However, autophagy plays a beneficial or detrimental role in neuronal death, depending on injury stage and degree of autophagy (Wei et al., 2012; Chen et al., 2014; Shi et al., 2021). Guo et al. (2021) conducted rat models of transient middle cerebral artery occlusion (tMCAO) and permanent MCAO (pMCAO), respectively. Then, the dynamic changes of autophagy activity, following tMCAO or pMCAO, were measured. The results demonstrated that both Beclin1 and LC3 expression levels, as indicators of autophagy, were significantly altered at different time points within seven days after tMCAO or pMCAO. Interestingly, autophagy induction elicited overt neuroprotection after tMCAO, and this effect was related to increased autophagy flux, indicating that autophagy plays a neuroprotective role, mainly at the subacute phase of tMCAO, but has few effects after pMCAO.

### Cerebral Ischemia and Disruption of Blood-Brain Barrier

After cerebral ischemia, the blood-brain barrier (BBB) not only has abnormal function but also suffers from structural damage (Abdullahi et al., 2018; Li et al., 2019; Sarvari et al., 2020). The microvascular basement membrane is interrupted or lost, as the components of the basement membrane (i.e., laminin fibronectin and type IV collagen) are significantly reduced. In addition, damage to the BBB is not only a destruction of anatomical structure, but also a selective compensatory mechanism (Sheikh et al., 2022). The connection between neurons and microvessels is not only related to regulation of blood flow but also to the permeability of BBB. Previous studies have demonstrated that the necessary anatomical structure for BBB permeability is the close connection between microvascular endothelial cells and astrocytes. Furthermore, the direct regulation of astrocytes on blood vessels is realized by promoting the expansion or contraction of blood vessels (Ito et al., 2011, 2013). Other studies have discovered that pericytes can migrate rapidly from microvessels during cerebral ischemia, suggesting that pericytes can play a similar role to glial cells in maintenance of BBB formation and function (Gonul et al., 2002; Duz et al., 2007; Al Ahmad et al., 2011). In addition, an increase of BBB permeability under pathological conditions is significantly correlated with destruction of the extracellular matrix (Yang et al., 2015; Michalski et al., 2020).

Matrix metalloproteinases (MMPs) are a group of zinc-dependent enzymes that have the ability to degrade the extracellular matrix. Under physiological conditions, MMPs are known to be involved in embryo development, angiogenesis, nerve development, and regulation of tissue remodeling (Parks, 1999; Vu and Werb, 2000; Page-McCaw et al., 2007). After the onset of cerebral ischemia, MMPs can be rapidly produced by neurons, astrocytes, microglia, and endothelial cells, leading to degradation and destruction of the matrix layer mucin, collagen fiber IV, and cellular fibronectin (Candelario-Jalil et al., 2009; Chaturvedi and Kaczmarek, 2014). Animals and patient studies have discovered that cerebral ischemia can induce expression of MMPs, particularly, the increased activity of MMP-2 and MMP-9, which is closely related to increased cerebral microvascular permeability, BBB destruction, inflammatory cell invasion, and brain edema (Rosell and Lo, 2008; Kurzepa et al., 2014; Yang and Rosenberg, 2015). In conclusion, MMPs degrade the major components of the extracellular matrix and cause damage to BBB, resulting in cerebral edema and transformational hemorrhage after ischemic stroke (Jin et al., 2010; Seo et al., 2012).

## Exercise and Restoration of Neurovascular Unit

The pathological process after the onset of cerebral ischemia is complex, and the substances and factors that are released post-stroke have different roles. Each component of the NVU becomes damaged after stroke and maintains the microvascular-neuronal connection. The integrity of NVU is key to the prevention and treatment of ischemic stroke. Exercise, as a neuroprotective method, has been validated to be involved in protecting NVU during ischemic stroke (Wang et al., 2014).

### Exercise and Blood-Brain Barrier Protection

After cerebral ischemia, a series of cascade reactions cause BBB dysfunction and an increase in cerebrovascular permeability, leading to brain edema. MMP-9 degrades the extracellular matrix, which is directly related to BBB dysfunction. Previous studies have indicated that MMP-9 reaches its peak at 48 h after ischemia, causing irreversible damage to BBB (Yang and Rosenberg, 2011). Inhibition of MMP-9 can alleviate dysfunction of the BBB, thereby reducing the degree of cerebral edema after cerebral ischemia (Chaturvedi and Kaczmarek, 2014; Kurzepa et al., 2014). Exercise can improve BBB function and integrity of the basement membrane after ischemia by inhibiting MMP-9 overexpression and upregulating tissue inhibitors of MMP (TIMP) (Guo et al., 2008a). Davis et al. (2007) discovered that exercise preconditioning ameliorates ischemic stroke-induced BBB dysfunction by strengthening the basal lamina with the involvement of MMP-9. Treadmill exercise can provide a protective effect on BBB disruption by degrading occludin, zonula occludens-1 (ZO-1), and an increase of MMP-9 after the onset of chronic cerebral hypoperfusion (Lee et al., 2017). Teymuri Kheravi et al. (2021) assessed the effects of two different exercises (i.e., swimming and treadmill training) prior to stroke induction. The results demonstrate that MMP-2 expression was increased after both exercise types, with reduced brain damage. In addition, exercise also alleviates BBB dysfunction by upregulating TNF-α levels. Furthermore, exercise training significantly increases the expression of TNF-α and alleviates BBB dysfunction, through extracellular-regulated protein kinase (ERK) 1/2 (Guo et al., 2008b; Chaudhry et al., 2010; Curry et al., 2010). Aquaporin (AQP)-4, an important regulator of cerebral edema formation, functions to alleviate cerebral edema in exercise preconditioning. In the study conducted by He et al. (2014), MRI was utilized to evaluate the dynamic impairment of cerebral edema after cerebral ischemia. The results demonstrate that treadmill pretraining improved the relative apparent diffusion coefficient (rADC) loss after cerebral ischemia, and that T2W1 values of the ipsilateral cortex and striatum decreased within 2 days after stroke, while the brain water content and expression of AQP4 were decreased at 2 days after ischemia following pretraining.

### Exercise and Neuronal Death

After the occurrence of an ischemic stroke, cell death, especially neuronal death, occurs in the core area of ischemia. Furthermore, the extent of infarction is related to the degree of interruption of blood supply. Reducing neuronal apoptosis can help improve the neurological function after the onset of cerebral ischemia (Memezawa et al., 1992; Li et al., 1998; Radak et al., 2017; Uzdensky, 2019).

Extracellular-regulated protein kinase 1/2 functions in the neuroprotective mechanism of exercise preconditioning. Lee et al. (2020) indicate that treadmill exercise improved short-term memory by inhibiting apoptosis in the hippocampus of the ischemia gerbils, which may be associated with the activation of the ERK-Akt-cAMP response element-binding protein (CREB)-brain-derived neurotrophic factor (BDNF) pathway. Similarly, swimming preconditioning improves the neurological outcome of cerebral ischemia in long-term ovariectomy rats, which is related to the activation of the ERK1/2/CREB/BDNF signaling pathway (Zhang et al., 2018). Zhou et al. (2018) discovered that the improvement of neurobehavioral performance by Willed movement training, following tMCAO, has been suggested to be involved in the ERK/CREB pathway. Notably, there was a region-specific discrepancy between ERK and CREB phosphorylation in a swim stress study (Shen et al., 2004). Exercise may also enhance the proliferation and differentiation of endogenous neural stem cells in the hippocampus of ischemia rats by enhancing phosphorylation of ERK. Subsequently, treatment with U0126 (an inhibitor of ERK) reversed the beneficial effects of exercise (Liu et al., 2018).

After the occurrence of ischemic stroke, heat shock protein (HSP)-70 (HSP-72) can protect neuronal apoptosis, caused by ischemia and hypoxia, and play an important role in cell survival and recovery. The increased synthesis of HSP-70 (HSP-72) can be utilized as an indicator of active repair and compensate for nerve reconstruction; it can also promote the recovery of neurological dysfunction after ischemia (Shevtsov et al., 2014; Kim J. Y. et al., 2018; Kim et al., 2020; Demyanenko et al., 2021). Recent studies have discovered that exercise can exert a neuroprotective effect on cerebral ischemia by regulating HSP-70 (HSP-72). Wang Y. L. et al. (2019) demonstrated that the percentage of HSP-72-containing neurons are tightly associated with a degree of ischemic stroke-induced brain injury, and exercise preconditioning can help improve neurological function post-stroke by preserving both the old and newly formed HSP-72-containing neurons. Liebelt et al. (2010) found that the expression of Bax and apoptosis-inducing factor (AIF) were reduced, while levels of Bcl-x(L) were increased in response to stroke after exercise. Additionally, inhibiting HSP-70 or pERK 1/2 reversed this resultant increase or decrease, leading to better neurological outcomes. However, inhibitors of phosphorylated ERK1/2 reduce brain injury, but do not reduce the expression of HSP-70, suggesting that ERK1/2 is not an upstream regulatory protein of HSP-70 after exercise preconditioning. A study of cerebral ischemia model induced by heat stroke showed that 3 weeks of pretreatment with progressive exercise-induced upregulation of HSP-72 and conferred neuroprotection against cerebral ischemia with reduced neuronal damage (Chen et al., 2007). Interestingly, poststroke exercise, if too early, can cause an elevated degree of cellular stress with increased expression of cell stress indicators, such as HSP-70 and hypoxia-inducible factor (HIF)-1α (Li et al., 2017b). The exacerbation of cell stress can be further aggravated during secondary brain injury post-cerebral ischemia, which indicates the importance of choosing an initiation time point of exercise and may have an effect on stroke recovery and rehabilitation (Li et al., 2017b).

Since the discovery of BDNF (Barde et al., 1982), its role in promoting neuronal regeneration and improving neurological functions has been widely studied. BDNF is mainly expressed in the hippocampus and cortex, and increasing the levels of BDNF to promote neuroregeneration is an important mechanism associated with the improvement of neurological function, after the onset of cerebral ischemia (Zhu et al., 2019, 2021; Tan et al., 2021). Studies have indicated that exercise can increase mRNA expression of BDNF in hippocampal regions of experimental animals, as well as improve hippocampal cell proliferation and cognitive function (Stagni et al., 2017; Liu and Nusslock, 2018). Many studies have sought to explore the mechanism underlying BDNF regulation of exercise-induced neuroprotection in ischemic stroke. Lee et al. (2020) demonstrated that treadmill exercise can attenuate memory impairment in cerebral ischemia gerbils by activating the ERK-Akt-CREB-BDNF signaling pathway. Another study that used swimming as the exercise method observed a similar phenomenon (Zhang et al., 2018). Caveolin-1/VEGF/BDNF and BDNF/TrkB signaling pathways are also involved in the recovery of motor and cognitive function after MCAO (Shi et al., 2016; Chen et al., 2019).

Numerous clinical and animal studies have suggested that regular aerobic exercise can increase the neuroprotective effect by enhancing the secretion of neurotrophins, including BDNF and insulin-like growth factor (IGF)-1, in the brain. Low concentrations of IGF-1 in the serum are found among chronic hemiparesis patients after they suffer from stroke (Silva-Couto Mde et al., 2014). In a randomized controlled trial, aerobic exercise, combined with cognitive training, improved the fluid intelligence of stroke patients. Upregulation with the higher serum IGF-1 expression was related to more robust improvements in cognition of patients, indicating that IGF-1 may participate in behaviorally induced plasticity (Ploughman et al., 2019). Ploughman et al. (2005) demonstrate that modest exercise can increase the IGF-1 expression, thereby contributing to the promotion of synaptic plasticity, resulting in an improvement of motor function after ischemic stroke. Exercise-induced IGF-1 can help protect cultured hippocampal cells against N-methyl-D-aspartate (NMDA)-mediated excitotoxicity by the negative feedback of NMDA receptor subunit NR2B (Li et al., 2017c). Zheng et al. (2014) discovered that an increase in physical exercise directly increases the number of neural progenitor cells by activating the IGF-1/Akt signaling pathway, thereby contributing to recovery of neurological function post-stroke. Similar results were observed by Chang et al. (2011).

### Exercise and Vascular Protection

Studies have demonstrated that exercise can protect the cerebrovascular system. Bullitt et al. (2009) discovered that aerobically active exercise contributes to the reduction of vessel tortuosity and elevation of small vessels, using the method of magnetic resonance angiography (MRA). A series of animal experiments reported that exercise led to an increase in the density of microvasculature and improvement in the blood supply of the brain. Monkeys, which are the closest human primates, show that 5 months of exercise can increase the number of blood vessels in the cerebral cortex. However, after 3 months of rest, the vascular density decreased to the baseline level, suggesting that only continuous exercise training maintains the promoting effect of angiogenesis (Rhyu et al., 2010). Ding et al. (2004) demonstrated that cerebral vascular integrity in the striatum region of middle-aged rats significantly improved after undergoing treadmill exercise training. Another study from the team discovered that integrins enhancement during exercise increased the neurovascular integrity post-stroke (Ding et al., 2006). Different durations of exercise have a similar effect. Four weeks of exercise increased the capillary distribution density and angiogenesis within the cerebellar region of rats (Isaacs et al., 1992). Additionally, 26 weeks of wheel running increased capillary and arteriole surface area densities and improved arteriolar reactivity and vasodilation in the motor cortex in response of exercise animals to hypercapsaic-hypoxic conditions (Stevenson et al., 2020a). Four weeks of voluntary wheel running demonstrated that sprouting angiogenesis is the main form of structural vascular plasticity that is detected in the motor cortex of rats, which is measured by vascular corrosion casts and resin replicas of the brain vasculature. In addition, increased capillary diameter and expanded endothelial cell nuclei diameters are observed in rats after exercise (Stevenson et al., 2020b).

Nitric oxide (NO) can help relax vascular smooth muscle cells and inhibit smooth muscle cell proliferation, which has an important function in inhibiting elevated blood pressure and vascular remodeling. Exercise can help stimulate phosphorylation and activation of AMP-activated protein kinase (AMPK) in vascular endothelial cells. This can promote phosphorylation of endothelial nitric oxide synthase (eNOS), increase the release of NO, improve endothelium-dependent relaxation function, and can be activated by shear force during exercise. Therefore, eNOS can increase NO production and enhance endothelium-dependent relaxation function (Lee-Young et al., 2009; Barr et al., 2017). Nitric oxide can be both protective and harmful in cerebral ischemia. NO is synthesized by nitric oxide synthase (NOS). Nitric oxide synthase can be divided into neuronal nitric oxide synthase (nNOS) and eNOS, both of which are expressed in the normal state, and inducible nitric oxide synthase (iNOS), which is expressed after injury. Studies have shown that eNOS has cerebral ischemia protection properties (van Faassen et al., 2009; Barr et al., 2017). Gertz et al. (2006) discovered that continuous exercise increases the levels of eNOS in the vasculature and improves the number of endothelial progenitor cells (EPCs) within the spleen and bone marrow. This boosts the circulating EPCs in the blood, thereby promoting neovascularization. Furthermore, animals that exercise have more newly generated cells in the vasculature, as well as a higher density of perfused microvessels, and increased blood supply of the brain in the ischemic region. However, after administration of NOS inhibitors or the antiangiogenic compound endostatin, the neuroprotective effect of exercise on angiogenesis and revascularization, as mentioned above, is reversed. This confirms that the improvement of outcomes and vascular function by exercise after stroke is related to the enhancement of angiogenesis and cerebral blood flow, partially through the eNOS-dependent signaling pathway. Exercise reduced brain injury following cerebral ischemia and restored impaired eNOS- and nNOS-dependent vascular function in type 1 diabetic rats (Arrick et al., 2012). Sun et al. (2019) indicated that exercise and exhaustive exercise animals both had higher expression of NO, increased NOS activity, and elevated expression of eNOS, nNOS, and iNOS. Interestingly, levels of eNOS are much higher within the exercise group, while iNOS has a significant increase in the exhaustive exercise group, which suggests that exercise can exert a neuroprotective effect by promoting levels of eNOS but not iNOS.

Vascular endothelial growth factor (VEGF) plays an important role during the process of exercise-induced angiogenesis. Exercise can induce the gene and protein expression of VEGF (Tang et al., 2010) and decreased brain infarct volume after the onset of cerebral ischemia (Ke et al., 2019). Measurement of the peri-infarct area of brain tissue shows an elevated expression of endothelial or angiogenesis markers (e.g., VEGF, VEGFR-2, and Ang-2), as well as endothelial progenitor cell marker (CD34+) after exercise (Pianta et al., 2019). The studies from Xie et al. (2019) indicated that exercise can alleviate neurological dysfunction after cerebral ischemia by promoting dendritic modification and synaptic plasticity, which is related to activation of caveolin-1/VEGF signaling pathway (Chen et al., 2019; Xie et al., 2019).

Infusion of endothelin-1 (ET-1), as a potent vasoconstrictor, into the animal’s hippocampus using stereotaxic surgery is a method that is frequently used to develop an ischemia model (Joo et al., 2012; Farokhi-Sisakht et al., 2020). A clinical study including 27 patients has shown that ET-1 expression increases in exercise-induced ischemia and has a prognostic value as a marker of ischemia severity (Lubov et al., 2001). In an endurance exercise study, athletes that exercised at an intensity of 130% of individual ventilatory threshold, and the plasma levels of ET-1 and ET-3, were measured. Interestingly, ET-3 increased faster than ET-1 after exercise (Maeda et al., 1997). Exercise preconditioning can increase levels of HIF-1α, trigger expression of ET-1, and elevated the release of brain natriuretic peptide (BNP) to cause vasodilation. This vasodilation involved the use of some other factors, including VEGF, which led to a restoration of brain blood flow and attenuation of ischemic injury (Wang H. et al., 2019). Furthermore, Zhang et al. (2014) also discovered that preconditioning exercise protected ischemic-induced injury by improving cerebral blood flow and regulating ET-1 expression.

## Conclusion

Exercise is an important method of preventing and rehabilitating cerebral ischemia, as it can not only reduce the incidence of stroke, but also play a protective role in the development of stroke, thereby reducing the severity and improving stroke outcome. Based on the current review, exercise can alleviate the damage of NVU after cerebral ischemia by inhibiting neuronal apoptosis, reducing BBB dysfunction, and promoting angiogenesis and synaptic plasticity. Elucidating the neuroprotective mechanism of exercise will be helpful in improving people’s understanding of exercise, and encourage patients that are at high risk of stroke to actively participate in exercise. They will also provide new therapeutic strategies and potential drug targets for the prevention and treatment of cerebral ischemia in the future. Many challenges and issues remain to be solved and emphasized in the further study, including, but not limited to, exploring optimal duration, initiating time, intensity of exercise, varying responses of different brain regions and cell types to exercise, and the discrepancy of gender and age in response to exercise. Another existing difficulty is the low long-term compliance of patients. Therefore, it is necessary to strengthen health education for the target high-risk population and improve their understanding and attention to the significance of exercise intervention.

## Author Contributions

HZ designed and conceptualized this review. JH drafted and revised the manuscript. HZ and QX edited the manuscript. All authors contributed to the manuscript and approved the final version.

## Conflict of Interest

The authors declare that the research was conducted in the absence of any commercial or financial relationships that could be construed as a potential conflict of interest.

## Publisher’s Note

All claims expressed in this article are solely those of the authors and do not necessarily represent those of their affiliated organizations, or those of the publisher, the editors and the reviewers. Any product that may be evaluated in this article, or claim that may be made by its manufacturer, is not guaranteed or endorsed by the publisher.
